# Foreign object detection in urban rail transit based on deep differentiation segmentation neural network

**DOI:** 10.1016/j.heliyon.2024.e37072

**Published:** 2024-08-28

**Authors:** Feigang Tan, Min Zhai, Cong Zhai

**Affiliations:** a School of Traffic and Environment, Shenzhen Institute of Information Technology, Shenzhen, 518172, China; b School of Civil Engineering & Transportation, South China University of Technology, Guangzhou, 510640, China; c College of Electronics and Information Engineering, Shenzhen University, Shenzhen, 518060, China; d School of Transportation and Civil Engineering and Architecture, Foshan University, Foshan, 528225, China

**Keywords:** Differential network, Foreign object detection, Deep neural network, Seam foreign object

## Abstract

With the increasing scale of urban rail transit, foreign object intrusion has become a significant operational safety hazard in urban rail transit. Although the laser-based automatic foreign object detection system has advantages such as long-distance detection and insensitivity to light changes, it has drawbacks such as large blind spots and low visualization. In response to the problems existing in laser detection systems, we proposed a novel video-based deep differentiation segmentation neural network for foreign object detection. Firstly, the foreign object detection is transformed into a binary classification problem, and the foreign object is determined as the image's foreground using image segmentation principles. Secondly, build a deep segmentation network based on deep convolution. Finally, perform morphological operations and threshold judgment on the foreground segmentation image to filter out the final detection results. To improve the detection effect, we reduced the impact of airflow disturbance by sampling and calculating the average background image. At the same time, the channel attention model and spatial attention model are added to the deep differentiation neural network. Collecting real data on subway platforms for experiments shows that the proposed method has a detection accuracy of 95.8 %, which is superior to traditional detection methods and recent image segmentation neural networks.

## 1 Introduction

**Fig. 1:**
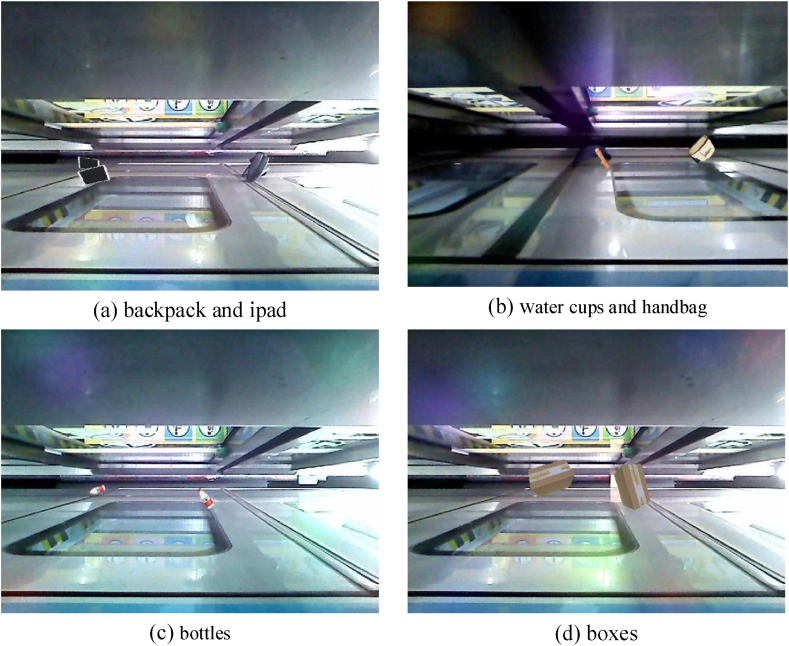
Example of foreign objects trapped in the gap between the platform and train doors.

Subway is the main transportation in modern cities. Its safety, punctuality, and comfort make it the first choice for people to travel by public transport [1]. Most subway stations have installed platform doors on the platforms to ensure the safety of passengers waiting for trains and enhance the comfort of the waiting environment. However, when the train stops at the platform, there is a narrow gap between the platform and the train doors. Passengers may have their belongings (such as backpacks and iPads in Fig. 1(a), water cups and handbags in Fig. 1(b and c), boxes in Fig. 1(d), and so on) left in the hole during boarding and alighting or caught in the gap when the train and platform doors are closed. These belongings or passengers stranded in the opening are foreign objects in the gap space, and they can pose a significant safety hazard to the subway operation. To solve the above safety hazards, many metro companies are studying the automatic detection of foreign objects in the gap space, especially unmanned driving technology [2], which is gradually moving towards application, making it a hot research topic in the metro industry [3,4].

The current automatic detection of foreign objects in the crevice space is mainly divided into laser or infrared sensor-based detection and image-based sensor detection [5]. The former has the defects of high installation requirements, high maintenance costs, and susceptibility to vibration, while the latter can better solve these defects and is favoured by researchers for its visualization and other features. The traditional foreign body detection methods are mainly frame differencing and background differencing [6], which have a fast detection speed but require that the two images involved in the comparison have no significant changes in the background area, resulting in low interference immunity [7]. To improve this problem, Barnich [8] used the vibe algorithm, and Charles [9] used the subsense algorithm to improve the detection accuracy. Although the traditional methods have gradually improved their performance during development, they are limited by the model, making their generalization, accuracy, and anti-interference ability still low. They can only cope with single-mode interference and cannot adapt to the complex and changing metro environment with light and background [10,11].

Long [12] proposed FCN lays down the basic semantic segmentation neural network structure, i.e., encoding-decoding structure. This structure extracts data features by encoding part, then classifies the features and recovers the size by decoding part to achieve the segmentation of pixels. After the proposal of FCN, it has received wide attention and improved and innovated on this basis. U-net [13] and SegNet [14] adjust the symmetry between encoding and decoding to enhance the segmented image. Especially, U-net's efficient, simple network and excellent segmentation performance make it widely used and innovative in the medical field [15,16]. The semantic segmentation technique based on deep learning can classify image pixels with strong generalization and anti-interference capability [17,18], which is suitable for the complex foreign object detection environment of the gap between the train doors and the PSD on the subway platform. In this scenario, foreign object detection of platform gap is transformed into a binary classification problem, i.e., foreground foreign object part and background non-foreign object part. Based on the above research status analysis, we proposed a novel differential deep neural network for foreign object detection between the train doors and the PSD on the subway platform.

The main innovation points of this paper are as follows: a novel foreign object foreground detection algorithm is proposed by combining the idea of difference contrast with a semantic segmentation method; a hybrid Gaussian background modelling of the image to be detected and the background image at the input of the network is performed to reduce the effects generated by airflow disturbance, vibration and light changes in the gap between the train door and the door of the subway platform.

## 2 Related work

At present, according to the composition of the system, the subway station platform screen door and the gap between the door's automatic detection of foreign objects can be divided into two categories: the physical characteristics of the automatic detection and automatic detection based on machine vision technology. The former [[19], [20], [21], [22]] (e.g., laser detection type, infrared light curtain type laser scanning type, etc.) have the disadvantages of large equipment size and low degree of visualization; while the latter [23,24] have the advantages of small equipment size, convenient installation and maintenance, high visualization degree, ease of expansion, etc. Compared with the former, the latter has become the main research direction in the field of rail transportation. Tan [5] designed an automatic detection system for foreign objects in the gap between platform screen doors and train doors to assist train drivers in performing pre-start safety checks. The target detection task based on image processing is mainly to find the target's position in the image and classify and identify it [25,26]. The current object detection algorithms are mainly divided into three categories: traditional object detection algorithms, deep neural network-based object detection algorithms, and image segmentation-based object detection algorithms. A brief overview of their research is as follows.

### 2.1 Traditional object detection algorithms

The traditional methods of object detection mainly include three stages: region selection, feature extraction, and classification. Region selection and feature extraction both require manual participation, and feature extraction is achieved by manually setting a sliding window size to extract features and cooperating with a classifier to achieve object detection. The detection results are easily affected by manual feature design, region selection, and other factors. This type of method has advantages such as low algorithm complexity and fast detection speed, but it has shortcomings such as low detection accuracy and weak robustness. Therefore, object detection algorithms based on deep neural networks and image segmentation have become the main research directions for object detection.

### 2.2 Object detection based on deep neural network

Deep convolutional neural networks can obtain an effective feature representation from the original pixels of the original image, which enables it to directly recognize objects based on human visual recognition rules from the original pixels of the image with the condition of minimal image preprocessing. Based on the deep convolutional neural network, it can be divided into two-stage target detection [27] and one-stage target detection [28]. The two-stage target detection algorithm generates a region, called a region proposal, and then classifies samples through a convolutional neural network. Common two-stage target detection algorithms include Mask R-CNN [29], SPPNet [30], Fast R-CNN [31] and Faster R-CNN [32]. Faster R-CNN has proposed a candidate area network (RPN) module, which greatly improves its accuracy and speed compared to R-CNN. However, it requires many samples and computing resources and is difficult to meet high real-time requirements in application scenarios. The one-stage target detection architecture uses DCNN to locate and classify directly. One-stage target detection can directly generate the target's category probability and position coordinates in one stage without generating candidate regions. Common one-stage target detection algorithms include SSD [33], DSOD [34], FCOS [35] and the YOLO series [[36], [37], [38]]. The YOLO series is an end-to-end target prediction algorithm based on global image information. As the latest achievement of the YOLO series, the YOLOv7 [39] target detection algorithm has higher detection accuracy and faster detection speed than previous models. By comprehensively benchmarking two types of object detection algorithms, it is not difficult to find that the stage object detection algorithm has the advantages of fast speed, simple and efficient algorithm, and easy construction. However, its detection accuracy still has room for improvement, and the detection effect for small and dense targets may not be as good as the two-stage algorithm.

### 2.3 Target detection based on image segmentation

Object detection based on image segmentation transforms object detection into a two-category problem: foreground image (i.e., detection object) and background image [40]. Image segmentation refers to judging its belonging category through the pixels of the picture. The traditional method mainly relies on the discontinuity and similarity characteristics of the intensity value in the image [41]. For example, the process based on edge detection uses its discontinuity to realize image segmentation according to the instantaneous change of intensity level or image grey level in the image, which mainly focuses on the identification of isolated points. Threshold segmentation or region segmentation, etc., segment images based on pixel similarity within a specific range according to preset criteria for image segmentation [42]. Therefore, traditional image segmentation methods can be roughly divided into threshold-based segmentation methods [43], region-based segmentation methods [44], cluster-based segmentation methods [45], and edge detection-based segmentation methods [46]. These methods guide segmentation by extracting low-level features of images, which have defects such as low precision and weak robustness.

The deep neural network simulates the human brain's learning process and automatically mines compelling features from large-scale data. The segmentation method based on deep learning overcomes the limitations of traditional manual feature segmentation, and its feature extraction performance is superior to conventional segmentation methods. It has become the mainstream method in the field of semantic segmentation, such as PSP-net [47], Deeplab series [48], etc. U-net is a segmentation network initially designed for medical image segmentation. It uses an encoder-decoder structure and skips connections to fuse shallow features and high-level semantics. Ding [49] proposed a dual-channel U-shaped network for image splicing forgery detection. The U-shaped network and its variants both use a single image as input, which can achieve good segmentation results under clear images and good lighting conditions. However, the gap environment between subway doors and platform doors is complex, with dark and varying lighting. A single image contains limited information and cannot cope with complex subway environments. Therefore, additional background information can be provided through background images to assist in foreground network segmentation.

High-quality and abundant labelled data is a crucial factor determining the learning quality of deep learning models. Without many labelled data sets, data augmentation is an effective way to solve this problem. In traditional data enhancement methods, typical methods are to rotate, translate, flip, crop, zoom, change intensity, suppress noise, etc., operations on the image, but such methods are prone to over-fitting. Migration learning uses the training parameters of the original model to initialize the new model, which can realize fast model training for limited label data. At present, there are mainly two methods based on transfer learning. One approach is fine-tuning the network model pre-trained on the ImageNet dataset; the other is transferring training data across domains: Kalinin [50] demonstrated that a network with an ImageNet pre-trained encoder outperforms a U-Net architecture trained from scratch in vascular hypoplastic lesion segmentation from wireless capsule endoscopy videos and robotic instrument segmentation from surgical videos.

## 3 Detection algorithm

### 3.1 Algorithmic framework

The foreign object detection in the gap between the subway platform door and the train door is a type of target detection applied in the subway platform, which is used to assist the train driver in completing the safety detection of the gap between the platform doors and the train doors before the train leaves the station, to ensure the safety of train operation [18]. Whether the existing subway platform is island type, side type, or mixed type, it may be a straight platform or a curved platform due to the influence of the platform topography. For a straight-line platform, the integrity of the light strip at the rear of the vehicle can be detected by the position of the front to determine whether there is a foreign object between the platform door and the vehicle door. For the curved platform, foreign object detection is carried out employing a relay or with each platform door as the detection unit.

After the train enters the station and stops in alignment, the gap between the platform door and the train door is in a foreign object-free state when the train door and the platform door are not opened [27], and the image is collected as the background image. The background image records the environmental state of the gap when both the train door and platform door are closed (e.g., light condition, train door position and form, sliding door position and shape, etc.), and these will assist subsequent images for foreground detection. Passengers get on and off the train after the doors and screen doors are opened, and when the passengers have finished getting on and off the train, the train driver will close the doors and platform doors afterwards. At this point, the gap space returns to the closed state before the doors are opened after the train enters the station and stops, but it is unknown whether foreign objects are in the gap. To prevent the gap between the doors and platform doors from being free of foreign objects and to ensure the safety of train operation, it is necessary to detect foreign objects in the gap between the doors and platform doors before the train leaves the station.

### 3.2 Average image

**Fig. 2:**
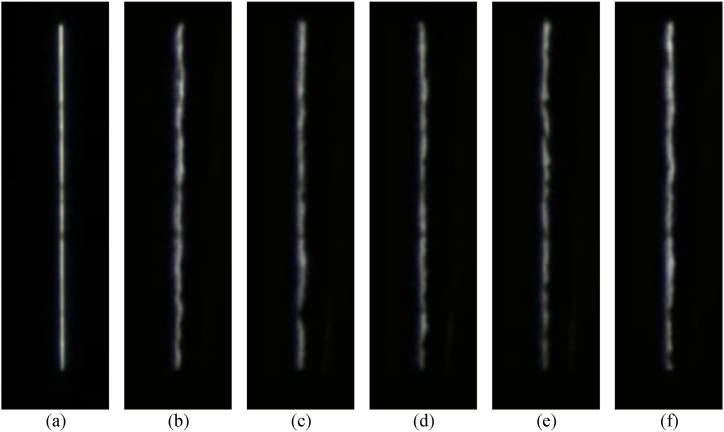
Schematic diagram of image acquisition in the complex aperture environment. (a) Indicates that the train has not entered the station light band image (subsequently called the original image) (b) to (f) show the train into the station after the golden band image. From the figure, it is easy to find that the complex environment (airflow disturbance, vibration, etc.) in the gap after the train enters the station makes the originally straight strip of lights become distorted and faint.

The train running in the subway tunnel will drive the surrounding airflow, especially after the train enters and stops at the station. The narrow gap between the train doors and screen doors is prone to airflow during train tunnel operation, as well as factors such as the temperature difference between the subway platform and the carriage after the train doors and PSD are opened. This causes significant airflow disturbance in the gap between the train doors and PSD, which in turn causes substantial interference in video image acquisition of the gap between the train doors and PSD. To facilitate the display of airflow disturbance, a camera was installed at the front of the platform car to capture the image of the light strip at the rear of the platform car. Fig. 2(a) shows the image of the light strip when it is not affected by air flow disturbance. while Fig. 2(b–f) is some extracted images where the gap between the platform door and the car door is affected by air flow disturbance and other factors.

After observing the dwell time of more than 100 trains entering the subway stations and researching train drivers, it is obtained that at least another 2 s pass after the train enters the station and stops in ATO driving mode before opening the doors and platform doors. At least another 10 s pass after the passengers finish getting on and off the train and close the doors and platform doors before starting the train to leave the station. Based on the above analysis, this paper constructs the background image and the image to be detected by sampling and averaging to cope with the complex gap environment. Suppose $P=\left[p_{1},p_{2},\ldots ,p_{1+nk}\right]$ is a set of image sequences denoting the (1 + *nk*) ^th^ idea in the image sequence. First, sampling is performed by the number of intervals *k*, and the extracted images are averaged as in Eq. (1) to obtain the background average map.

(1) $$P^{\prime }=\frac{1}{n}\sum_{i=0}^{n}p_{1+ik}$$

**Fig. 3:**
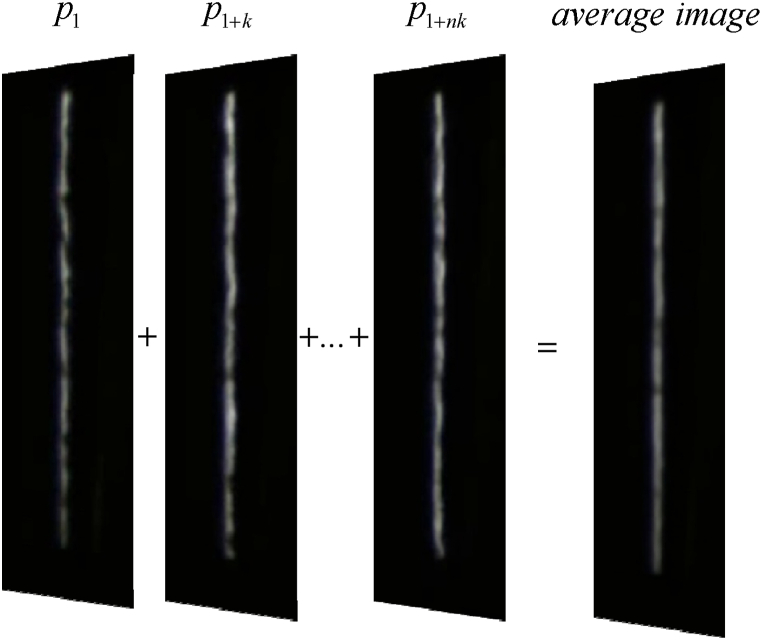
The schematic diagram of average image generation.

Fig. 3 shows the process of generating an average image $P^{\prime }$ with a sampling interval of *k*, and the resulting intermediate image $P^{\prime }$ resembles the typical standard image in Fig. 2 (a). Therefore, after the sampling and averaging process, the effect of factors such as airflow disturbance in the slit can be effectively suppressed.

### 3.3 Deep differentiation segmentation neural network

UNet is an efficient and concise network, and semantic segmentation networks have a relatively symmetrical encoding decoding structure, which improves image segmentation accuracy by integrating feature map information at different scales. Due to its excellent performance, it is widely used in the field of medical image segmentation [15,16]. FlowNet adopts a dual input and correlation structure, which can effectively infer more accurate information based on the correlation information between two images. Attention models based on attention mechanisms have been widely applied in artificial intelligence-related fields such as natural language processing, statistical learning speech recognition, and computer vision. The visual attention mechanism is a natural ability of the human brain. For example, when we see an image, we first quickly scan the image and then focus our attention on the target area with bright colours, clear edges, or protrusions for further observation. At the same time, in the process of edge computing, due to the limitation of computing resources, to make better use of the limited computing resources, we need to apply the limited computing resources to the target areas of real concern, and the attention mechanism can allocate computing resources to more important tasks.

**Fig. 4:**
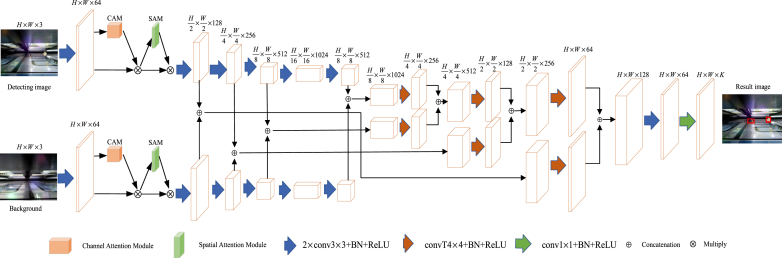
The DDSN network structure.

Based on the above analysis, the double-layer U-shaped network is simple and efficient, and the attention mechanism can help the network focus on key target candidate regions. We want to integrate their advantages to achieve more efficient detection of foreign objects in the gap between subway platform doors and platform doors. Therefore, we have designed a novel foreground detection network called Deep Differential Segmentation Network (DDSN), whose network structure is shown in Fig. 4. Introduce the channel attention model and spatial attention model in the encoding structure. Compared with UNet, DDSN extracts features of different scales from two images through parallel processing, and associates features of the same scale by position. This association enables the network to infer foreground pixels based on the intrinsic feature information and comparative differences between the two images.

The DDSN network consists of two parts: encoding and decoding. The encoding part takes both the detected and background images as inputs and extracts their features using the same network parameters. After obtaining a series of feature maps of different scales, the feature maps of the same scale in the two images are connected to associate their features. The decoding part gradually up-samples the associated feature maps at different scales to recover the size and obtain the foreground segmentation map. The up-sampling is performed by transposition convolution, starting from the feature map with the most miniature scale. The results obtained after each up-sampling are cascaded with the feature information of the previous scale (as shown in Eq. (2) and then up-sampled again to enable the decoding part to analyze and infer the results based on the feature information at different scales. In the up-sampling process, the number of channels of the feature map is reduced while increasing its ranking to minimize information loss. In the multi-scale feature fusion process, further features are extracted from the correlated feature maps using a convolutional layer and then connected to the up-sampled feature maps, thus making the process more learnable.

(2) $$F_{n}=\left[f_{n}^{b},f_{n}^{d}\right]$$

Where *n* denotes the *n*th layer in the network structure, $f_{n}^{b}$ and $f_{n}^{d}$ represents the background image and detection image features in the *n*th layer of the network structure, respectively.

### 3.4 Loss function

Foreground detection is a binary classification problem, i.e., foreground and background. At the same time, the background area in the image generally occupies a relatively large space. It causes an unbalanced foreground and background distribution (similar to negative sample distribution, much higher than favourable sample distribution). Therefore, this paper uses Binary Cross Entropy Loss (BCEL) and Focal Loss (FL) for linear combination to obtain the loss function *L*, as shown in the following Eq. (3):

(3) $$L=\alpha L_{bcel}+\left(1-\alpha \right)L_{fl}$$

Where $L_{bcel}$ represents *BCEL*, whose calculation formula is shown in Eq. (4), and $L_{fl}$ represents *FL*, and its calculation formula is shown in Eq. (5), and α is the weight parameters, in general, we have α∈[0,1].

(4) $$L_{bcel}=\left\{ \begin{array}{c} -\log\;p\left(x\right),y=1\\ -\log\left(1-p\left(x\right)\right),y=0 \end{array}\right.$$

(5) $$L_{fl}=\left\{ \begin{array}{c} -\beta {\left(1-p\left(x\right)\right)}^{\gamma }\;\log\left(p\left(x\right)\right),y=1\\ -\left(1-\beta \right)p\left(x\right)\log\left(1-p\left(x\right)\right),y=0 \end{array}\right.$$

Where: y and p(x) are the label value and the predicted value, β is the positive sample weight coefficient, the β∈[0,1], and γ is the modulation coefficient. Based on the literature [28] and experimental tests, each parameter is defined as α=0.65, β=0.98, γ=0.2.

### 3.5 Foreign objects detection

After obtaining the foreground segmentation map, we use the global threshold method (as shown in Eq. (6)) to classify the foreground and background to accept foreground candidate regions. Where *res* = 1 denotes foreground and *res* = 0 denotes ground, f(x,y) denotes the pixel value size of the foreground segmentation map at that point, T is the threshold value, and *T*_1_ = 0.56 is set in this paper.

(6) $$res=\left\{ \begin{array}{c} 1,if\;f\left(x,y\right)>T_{1}\\ 0,\text{otherwise} \end{array}\right.$$

Due to factors such as lighting, there may be some noise in the foreground segmentation image obtained from the above equation. Considering that foreign objects in the gaps have a certain volume, mapping them to the image has a certain area size. Therefore, after obtaining the foreground candidate regions, we use morphological closure operations to eliminate some interference noise and then perform foreign object detection according to Eq. (7). Where obj=1 denotes objects in the gap, while obj=0 signifies nothing in the hole, ϕ is the minimum area of the foreground connected domain, and $T_{2}$ is the judgment threshold. According to the field foreign matter test, the minimum area of foreign matter connected domain is calculated as 200. The confirmation process for foreign object detection is shown in Table 1.

(7) $$obj=\left\{ \begin{array}{c} 1,if\;\phi <T_{2}\\ 0,otherwise \end{array}\right.$$

**Table 1:** The confirmation process for foreign object detection.

step1:	Calculate the format *n* of the foreground foreign object candidate region after morphological operations.
step2:	Initialize calculator *i*, determine threshold *t*, and determine the minimum area *s* of the foreground connected domain.
step3:	***while****i* < *n****do***
step4:	*s*[*i*] = get_min_area(i)
step5:	***if****s*[*i*] < $T_{2}$***then***
step6:	obj[*i*] = 0
step7:	***else***
step8:	obj[*i*] = 1 ***end if***
step9:	i←i+1, and go to step 3 until completes the whole process
step10:	***end while***

## 4 Experiments

### 4.1 Datasets brief

In this experiment, with the cooperation of a certain subway company, a camera was installed on the platform door of a certain station, and data was collected from a top-down perspective on the gap between the car door and the platform screen door. The camera is about 2.3 m away from the platform ground, with a resolution of 640x480 and a frame rate of 30fps. Simulate foreign objects for data collection by placing common and potentially left objects such as backpacks, umbrellas, hats, and water bottles in the gaps. A total of 158 sets of sample data were collected on-site. To better evaluate the algorithm, this article augments the data by cropping foreign objects and randomly pasting them in the gap area. The original 158 sets of data are expanded to 1600 sets, with 1400 sets of samples as the training set and 200 sets of samples as the testing set.

### 4.2 Experimental parameters

The experimental computer parameters are CPU: Intel(R) Core(TM) i5-9400F; Memory: 16 GB; GPU: NVIDIA GeForce GTX 1650; OS: windows 11. software environment: python 3.9, PyCharm 2020.1, torch 1.9.1+ cu111. We use precision rate P, recall rate R, and reconciliation mean F as evaluation indexes to evaluate the experimental results objectively, specifically, it corresponds to Eq. (8), Eq. (9) and Eq. (10) as follows.

(8) $$P=\frac{N_{TP}}{N_{TP}+N_{FP}}$$

(9) $$R=\frac{N_{TP}}{N_{TP}+N_{FN}}$$

(10) $$F=\frac{2\times P\times R}{P+R}$$

Where $N_{TP}$ represents the number of positive samples that are correctly classified, $N_{FP}$ the number of positive models that are incorrectly typed, and the number of negative samples that are incorrectly classified.

### 4.3 Ablation experiments

#### 4.3.1 The attention mechanism

**Fig. 5:**
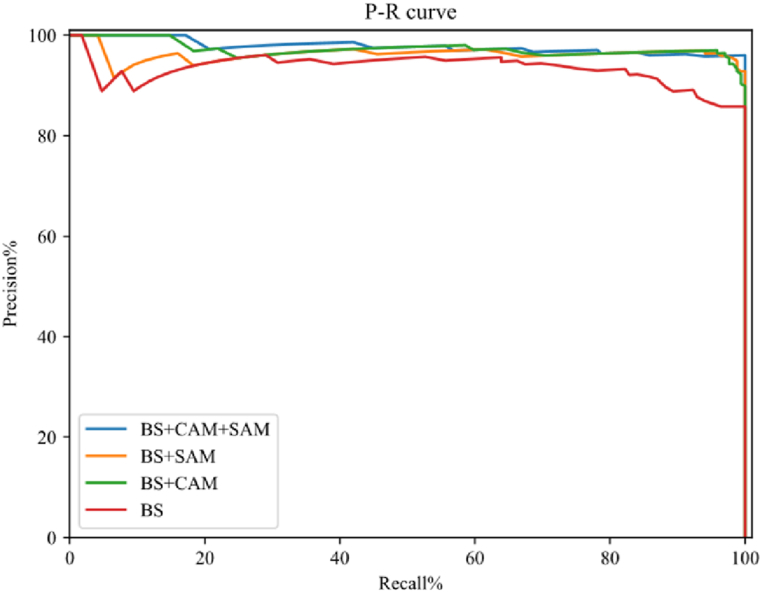
P–R cure of ablation experiments.

We validated the effectiveness of adding attention models in network structures by designing ablation experiments. According to the principle of variable uniqueness in ablation experiments, we designed the BS + x method based on a double-layer U-shaped network without an attention model (i.e. basic structure (BS)), where x is the variable and is designed as channel attention (CAM), spatial attention (SAM), channel attention, and spatial attention (CAM + SAM), respectively. The experimental comparison results are shown in Fig. 5. It is evident that adding the attention model improves the detection results, especially when adding both the channel attention model and the spatial attention model improves the detection compared to the primary network.

#### 4.3.2 The background reference image

**Fig. 6:**
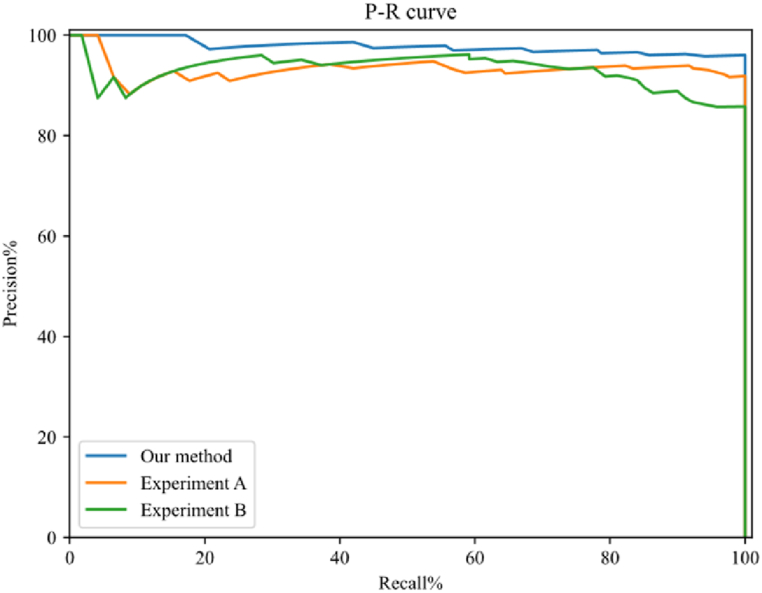
Horizontal comparison of P-R curve of ablation experiments under different methods.

To further test whether the background image has any effect on image segmentation, we set up the following two experiments for ablation experiments. Experiment A: The overall network structure remains unchanged, and the background image in the network is changed to the corresponding image to be detected. Experiment B: Considering that the background image removal cannot be applied to the original network structure, we change the original network structure to adapt to the no background image case. Firstly, the part of the original network structure related to the background image is removed. Then, after the features of the image to be detected are extracted in the encoding part, the feature map is directly inputted into the decoding part for subsequent calculations. Due to the lack of background image feature connections, the convolution kernel channel and the number of each convolutional layer in the decoding region are reduced by half to ensure the network can operate normally. A comparison of the P-R curves of the experimental results is shown in Fig. 6. It is easy to see that the P and R metrics of both Experiment A and Experiment B have decreased. Experiment A did not change the network structure, but its lack of background image reference caused its accuracy to fall slightly. The network structure of experiment B was altered, and its accuracy decreased more compared to that. This shows that inputting background images into the network helps to improve network judgment.

### 4.4 Comparison with traditional methods

The traditional foreign body detection methods mainly include the frame difference method, the hybrid Gaussian model [6], and Vibe [8]. In this paper, we select the above three modes and the proposed method for comparison experiments, and the results are shown in Table 2. As shown in the table, the proposed method in this paper is substantially ahead of the other accuracy methods. Since the traditional methods are artificially designed features, they are weakly robust to light variations. However, the illumination in the slit is easily affected by the number of passengers in the train and platform, and the airflow disturbance in the slit also affects the illumination variation. Our method uses a deep neural network and introduces an attention model to better overcome light and airflow disturbance.

**Table 2:** Experimental results compared with traditional methods.

	*P*/%	*R*/%	*F*/%
Frame difference	12.3	19.8	15.2
GMM	13.2	30.0	18.3
Vibe	12.0	19.5	14.8
Our method	95.3	88.6	91.8

### 4.5 Comparison of different network methods

**Fig. 7:**
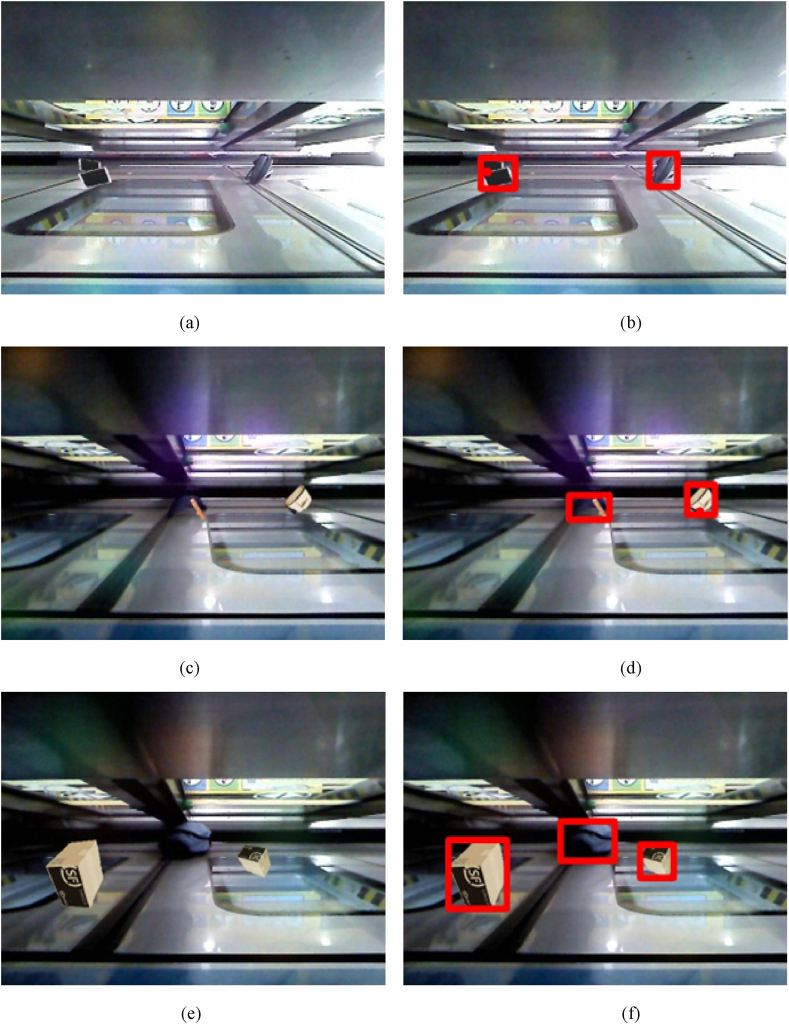
Schematic diagram of partial test results: where (a), (c) and (e) are the detection images, and (b), (d) and (f) are the detection results.

The method proposed in this article is based on detecting foreign objects as the foreground of an image with the background image as a reference, and its principle is similar to image segmentation. The YOLO series is a mainstream algorithm for object detection based on deep convolutional neural networks [36], which has been widely studied and achieved good results in the field of rail transit [38,39]. YOLOv7, as a representative of this series, has excellent detection performance [37]. Therefore, this article chooses four image segmentation-based network methods: UNet [13], SegNet [14], DifferentNet [7], and YOLOv7 [37] based on deep convolutional neural network methods for comparative experiments to verify the effectiveness of the algorithm proposed in this article. The comparison results are shown in Table 3. Since the first three methods are single-image input networks, only the detection images are trained and tested during the experimental process. YOLOv7 has achieved very good results in object detection and recognition, as we use a top-down approach to detect foreign objects left by passengers in narrow gaps. The detection effect is poor due to changes in lighting at the gaps, foreign object shapes, and changes in the perspective of the foreign object being photographed. DifferentNet is a two-image network, and both the detection and background images are used for training and testing. The experimental results show that the UNet-based transformation network performs better than the other networks but is slightly inferior to the method in this paper. This is mainly because the attention model is added to the network structure in this paper, while it is more profound in the coding layer than it is. Fig. 7(a–c, e) is the original detection image, including iPad, backpack, box, etc. while Fig. 7(b–d, f) are the corresponding detection results respectively.

**Table 3:** Experimental results compared with different network methods.

	*P*/%	*R*/%	*F*/%
UNet [13]	72.6	71.9	72.3
SegNet [14]	78.7	69.5	73.8
DifferentNet [7]	92.1	87.0	89.5
YOLOv7 [37]	83.3	75.0	78.9
Our method	95.3	88.6	91.8

## 5 Conclusion

In this paper, the detection of foreign objects in the gap between subway platforms' doors and platform doors is transformed into a binary classification problem: the gap image foreground and background image segmentation problem. We use three aspects to improve the detection effect of the gap between the doors and platform doors of subway foreign objects. First, the average image is calculated by sampling to reduce the influence of factors such as airflow disturbance between gaps. Second, the classical U-shaped neural network is improved, and a background reference image is introduced to enhance the segmentation effect. Third, the attention mechanism is introduced to increase the attention to the foreground objects in the image to improve the detection effect. By designing relevant experiments for verification, it can be seen from the experimental results that the Deep Segmentation Network proposed in this paper has a better detection effect than other current methods. Efficient, convenient, safe, and other characteristics of the real-time algorithm's subway operation and the rigorous requirements' reliability, while the current experimental sample size and the types of foreign objects are minimal. In addition, the number of passengers on the platform and in the car will also impact the environment around the gap, such as lighting. These shortcomings will be our next research focus.

## Data availability statement

This research was not applicable, no data was used for the research described in the article.

## CRediT authorship contribution statement

**Feigang Tan:** Writing – original draft, Methodology, Conceptualization. **Min Zhai:** Visualization, Software, Methodology, Investigation, Formal analysis. **Cong Zhai:** Writing – review & editing, Supervision, Funding acquisition.

## Declaration of competing interest

The authors declare that they have no known competing financial interests or personal relationships that could have appeared to influence the work reported in this paper.
